# Visualization and quantification of coconut using advanced computed tomography postprocessing technology

**DOI:** 10.1371/journal.pone.0282182

**Published:** 2023-02-24

**Authors:** Shenghuang Lin, Yu Zhang, Li’an Luo, Mengxing Huang, Hongxing Cao, Jinyue Hu, Chengxu Sun, Jing Chen

**Affiliations:** 1 Haikou Affiliated Hospital of Central South University Xiangya School of Medicine, Haikou, China; 2 College of Computer Science and Technology, Hainan University, Haikou, China; 3 Siemens Healthineers, Guangzhou, China; 4 College of Information and Communication Engineering, Hainan University, Haikou, China; 5 Coconut Research Institute, Chinese Academy of Tropical Agricultural Sciences, Wenchang, Hainan, People’s Republic of China; William & Mary, UNITED STATES

## Abstract

**Introduction:**

Computed tomography (CT) is a non-invasive examination tool that is widely used in medicine. In this study, we explored its value in visualizing and quantifying coconut.

**Materials and methods:**

Twelve coconuts were scanned using CT for three months. Axial CT images of the coconuts were obtained using a dual-source CT scanner. In postprocessing process, various three-dimensional models were created by volume rendering (VR), and the plane sections of different angles were obtained through multiplanar reformation (MPR). The morphological parameters and the CT values of the exocarp, mesocarp, endocarp, embryo, bud, solid endosperm, liquid endosperm, and coconut apple were measured. The analysis of variances was used for temporal repeated measures and linear and non-linear regressions were used to analyze the relationship between the data.

**Results:**

The MPR images and VR models provide excellent visualization of the different structures of the coconut. The statistical results showed that the weight of coconut and liquid endosperm volume decreased significantly during the three months, while the CT value of coconut apple decreased slightly. We observed a complete germination of a coconut, its data showed a significant negative correlation between the CT value of the bud and the liquid endosperm volume (y = −2.6955x + 244.91; R^2^ = 0.9859), and a strong positive correlation between the height and CT value of the bud (y = 1.9576 ln(x) −2.1655; R^2^ = 0.9691).

**Conclusion:**

CT technology can be used for visualization and quantitative analysis of the internal structure of the coconut, and some morphological changes and composition changes of the coconut during the germination process were observed during the three-month experiment. Therefore, CT is a potential tool for analyzing coconuts.

## Introduction

Coconut is an important tropical food and oil raw material, its medicinal, ornamental and other utilization value has been widely recognized. for example, coconut oil is widely accepted to have beneficial effects on cardiovascular disease [1, 2]. Studies have suggested that coconut oil has health benefits for individuals with high blood sugar levels, inflammation, and obesity [3, 4]. Observation and analysis of the structure and composition of coconut may be important for nutritional analysis or seed selection.

Coconut is large, dry drupe, ovate in shape. The exocarp is green or yellow, turning to brown, depending on cultivar and maturity. The mesocarp is fibrous and dry at maturity. The endocarp is the hard outer shell that surrounds the seed. The seeds are the largest of all plants and have a thin brown seed coat. The seeds are filled with the endosperm, which is either solid and attached to the seed coat, called “solid endosperm”, or liquid, called “liquid endosperm”. The seed has 3 germ holes, making the seed look like a miniature bowling ball. Two holes are usually blocked and the embryo is located below the third functional hole. The embryonic root and shoot emerge from functional pores. And the cotyledon forms a spongy mass of tissue within the seed cavity (called “coconut apple”) that absorbs the endosperm and fuels initial growth [5, 6]. However, it is difficult to observe the internal structure or growth process of coconuts due to its thick mesocarp and hard endocarp.

In several studies on the structure of coconuts, either in vitro tissues of coconuts were obtained for observation and analysis at the microscopic level [7, 8], or the medicinal value of extracts isolated from coconuts was studied [9]. Most studies still cut coconuts to observe their macroscopic morphological structure. Analyzing the internal three-dimensional (3D) morphological structures of coconut without damage is difficult. Splitting coconut for analysis results in the destruction of the coconut structure, which makes it impossible to perform subsequent research. For example, observation and measurement of the internal structural changes of coconut during germination, including coconut water, endocarp, embryo, and coconut apple, are difficult using existing tools. Additionally, the process of seed selection in coconuts is difficult, because whether the embryo is alive or not cannot be confirmed based on its appearance alone.

Computed tomography (CT) is a kind of fluoroscopic imaging technology. Its imaging principle is that the turb emits X-rays under high voltage. The X-rays pass through the object and then attenuate and finally reach the detector. The tube rotates and scans around the three-dimensional object, and the detector can obtain cumulative attenuation signals from different angles, so as to reconstruct the CT image of the object layer by layer, which present the spatial distribution of X-ray attenuation of the object. Each pixel value in the CT image represents the X-ray attenuation of the object at this position, which is called CT value [10]. Traditional CT images are gray-scale two-dimensional cross-sectional images. With the development of post-processing technology, multi-angle 2D reconstruction (called multiplanar reformation (MPR)) and 3D reconstruction (called volume rendering (VR)) can be realized using volumetric CT data. The VR technology can reconstruct various 3D model of object structure by giving different transparency and color to the spatial point based on CT values, which is of great significance for understanding the spatial relationship of different structures. CT has been widely used in medicine and food industry. In the medical field, for example, the VR can provide accurate information about the location and size of lesions, making it easier for surgeons to identify tiny target areas during surgery [11]. Using MPR and VR techniques to measure morphological parameters such as lung compression ratio or airway alterations, has been proved to be useful in the diagnosis and improve the clinical work efficiency [12, 13]. Furthermore, both techniques could detect further bone fractures in body parts difficult to investigate during autopsy (i.e. posterior regions), facilitating the pathologist in the reconstruction of events and in determining the cause of death in evaluating trauma victims [14]. In addition to improving diagnostic accuracy, planning, follow-up and clinical decision making for complex clinical cases, a series of cases illustrate the value of VR and MPR techniques in teaching and research [15–18]. In agricultural applications, some researchers explored the value of X-rays in plant observation and used CT to scan opaque soil blocks to obtain 3D data of plant roots and visually analyze root growth [19–21].

In this study, we explore the feasibility of using medical high-resolution CT and 3D postprocessing technology to observe and visualize the internal structure of the coconut and the germination process without damage.

## Materials and methods

This study was approved by the institutional review board of Haikou Affiliated Hospital of Central South University Xiangya School of Medicine.

### The general materials

On April 9, 2021, 12 brown coconuts aged 12 months were taken from two coconut trees in Leiming Town, Ding’an County, Hainan Province. Each coconut tree has old and tender fruits, and the space between the two trees is about four meters. Coconut trees are approximately 25 years old, with a tree height of more than 10 meters and approximately 32 leaves.

**Fig 1:**
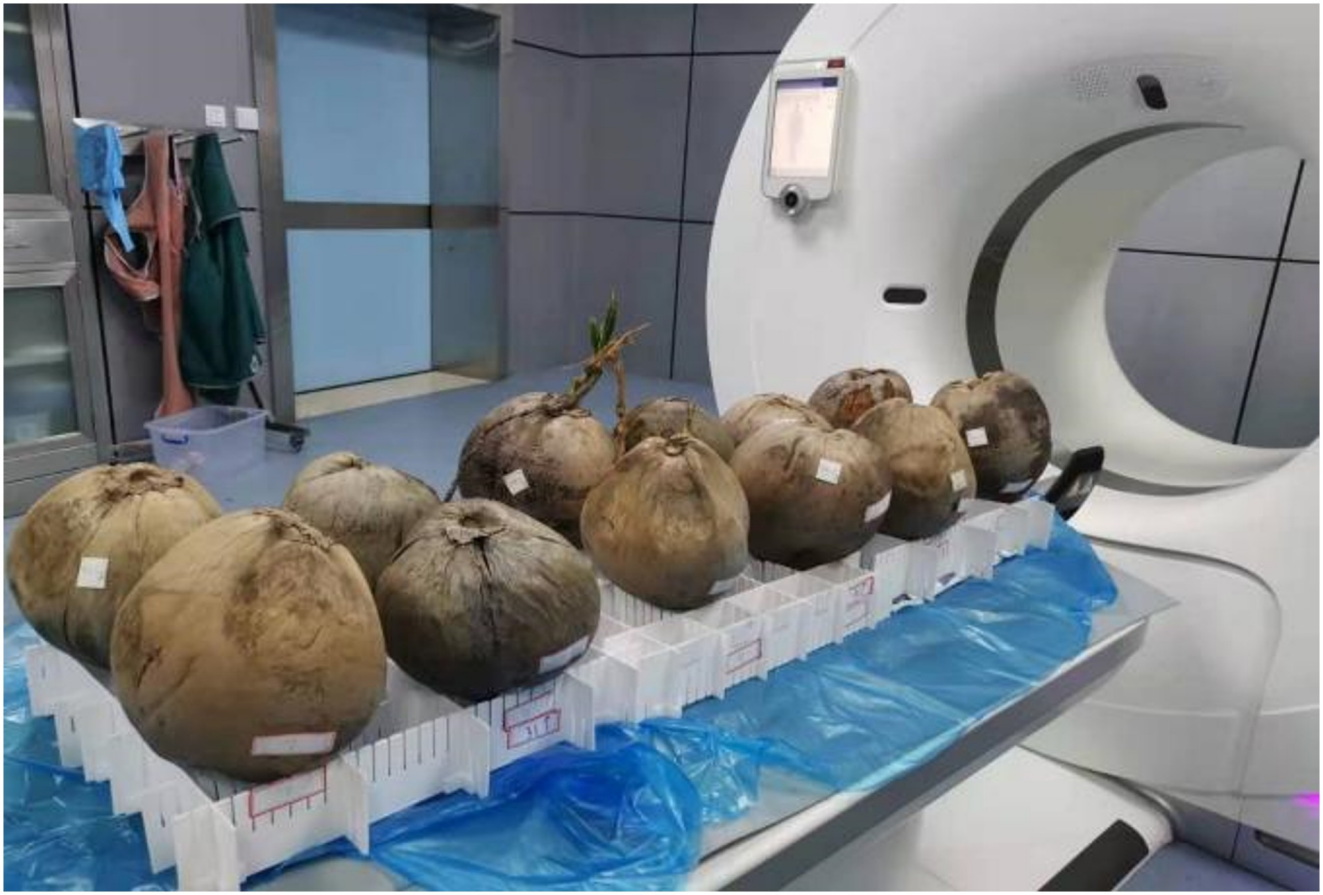
The position of the coconuts on the computed tomography scanner.

Among the 12 coconuts, one was sprouting on the outside, whereas the remaining 11 were not. Before each scan, the weight of each coconut was measured using a weighing balance and recorded. Three sets of porous plastic films were used to fix the 12 coconuts, four for each set, so that the coconuts were kept in the same horizontal plane. Coconut experiment process from April to July 2021. Coconuts were placed in natural air for storage and observation, the temperature was 24°C~34°C, the humidity was 70~ 89%. The twelve coconuts were placed in six rows and two columns on the mold for scanning (Fig 1).

### Image acquisition

This study was conducted using a dual-source CT scanner (SOMATOM Definition Flash, Siemens Healthineers, Erlangen, Germany). The CT scan parameters were as follows: slice thickness/increment = 0.6 mm/75%, tube voltage = 120 kv, tube current = 250 mAs, field of view = 400 × 400 mm, and frame speed = 0.5 s/rev.

In each experiment, the coconuts were uniformly placed with their top part facing up and the bottom part on the fixing film (Fig 1). According to the anatomical position standard for human scanning, the right-hand column was placed from the head to the foot as numbers 1–6, and the left-hand column was placed on the same right-hand side as numbers 12–7. Numbers 1 and 12 are the positions of the human head, and numbers 6 and 7 are the positions of the human feet. Additionally, the fixed mold and coconut were marked using a marker to ensure the unity and integrity of scanning data in the later period.

### Image processing

The gray scale CT images were generated and uploaded to the Siemens Syngo.via postprocessing workstation, and multiplanar reformation (MPR) and volume rendering (VR) were created. Gray-scale axial CT images were used to observe the internal structure of coconut layer by layer, delineate ROI on the location of exocarp, mesocarp, endocarp, embryo, bud, solid endosperm, liquid endosperm, and coconut apple to measure CT values. The MPR technique can obtain two-dimensional image from different angles, which can be used to observe the internal structural morphology of coconut from a cross-sectional perspective. In addition, morphological parameters such as the diameter of three pores on the endocarp and the height of the bud could be measured by adjusting the angle of MPR image to the section of the structure with the clearest display and the largest area. The VR technique can reconstruct various 3D models to visualize the different structures inside the coconut by giving specific transparency and color to the voxel point based on CT values. We have empirically developed many models for the visualization of coconut structures. The volume of the structure is obtained by creating a specific VR model of the structure and then calculating the voxel volume.

### Statistical analysis

All statistical analyses were performed using SPSS (version 25, IBM Corp). The CT values and morphological parameters of different coconuts structures were measured and statistically analyzed. The distribution of CT values of different structures was counted. Moreover, the changes in the CT value and morphological parameters of each structure over time were analyzed with analysis of variances (ANOVAs) for temporal repeated measures, which was conducted with four time point data at a four-week interval. The correlation between CT and morphological parameters were analyzed, and regression analysis was performed for those parameters with significant correlation.

## Results

**Fig 2:**
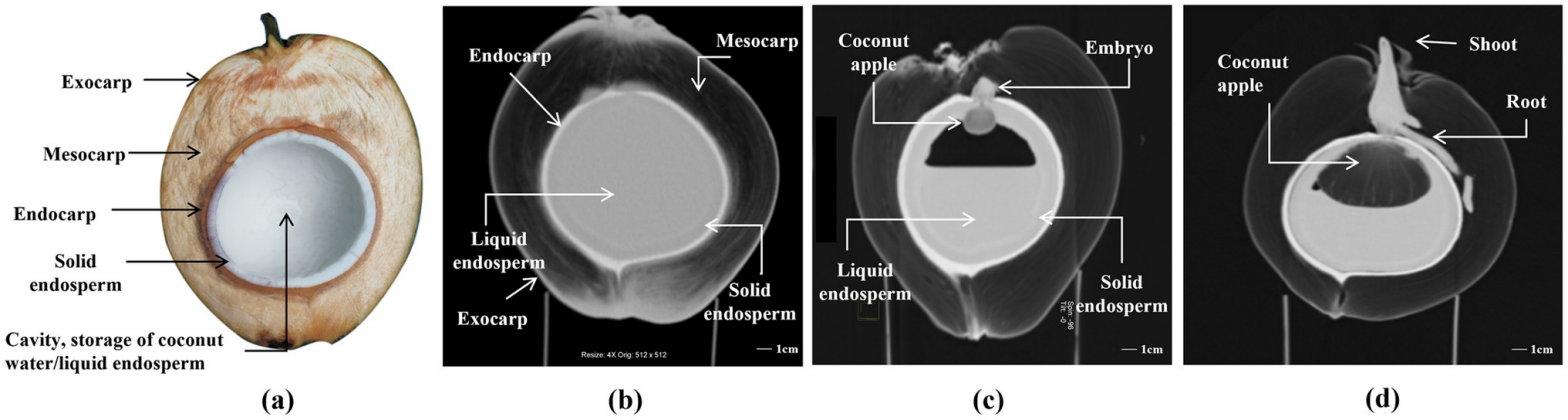
A physical photo of cut coconut and three computed tomography multiplanar reformation images of different coconuts.

**Fig 3:**
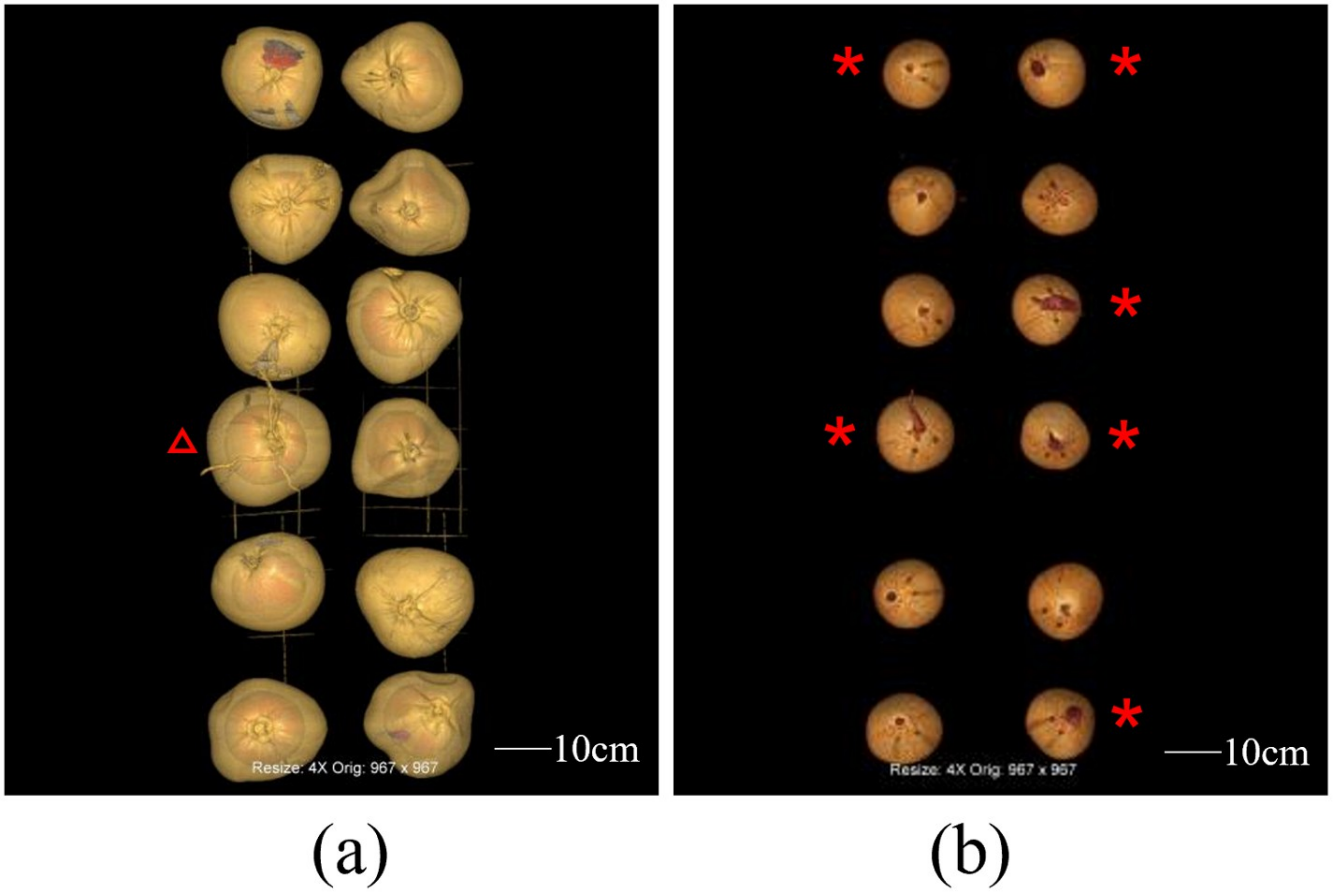
Different volume rendering models for displaying the overall condition of the coconuts in the first scan. (a) The exocarp of the coconut, with a coconut sprout visible on the exocarp (red triangle). (b) The endocarp of coconuts, bud merged from the largest pore (red asterisk).

**Fig 4:**
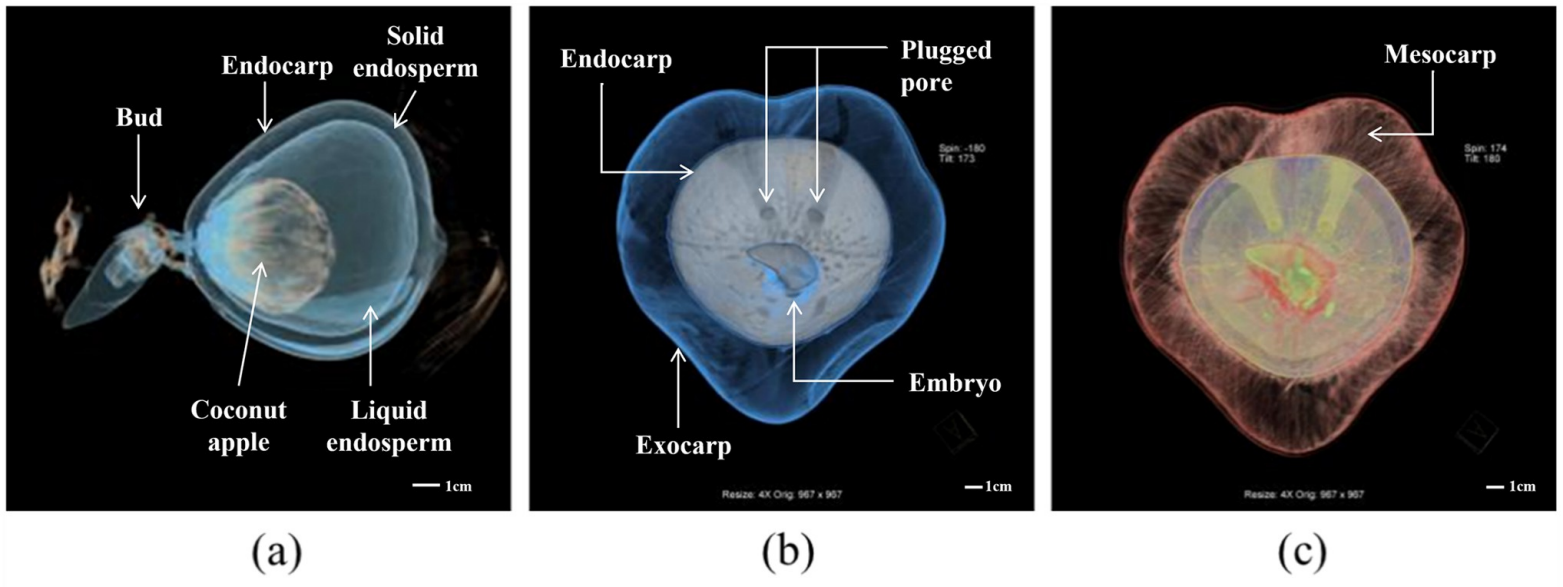
Different volume rendering models for displaying different structures on one coconut: Exocarp, mesocarp, endocarp, coconut apple, endosperm, and buds.

**Fig 5:**
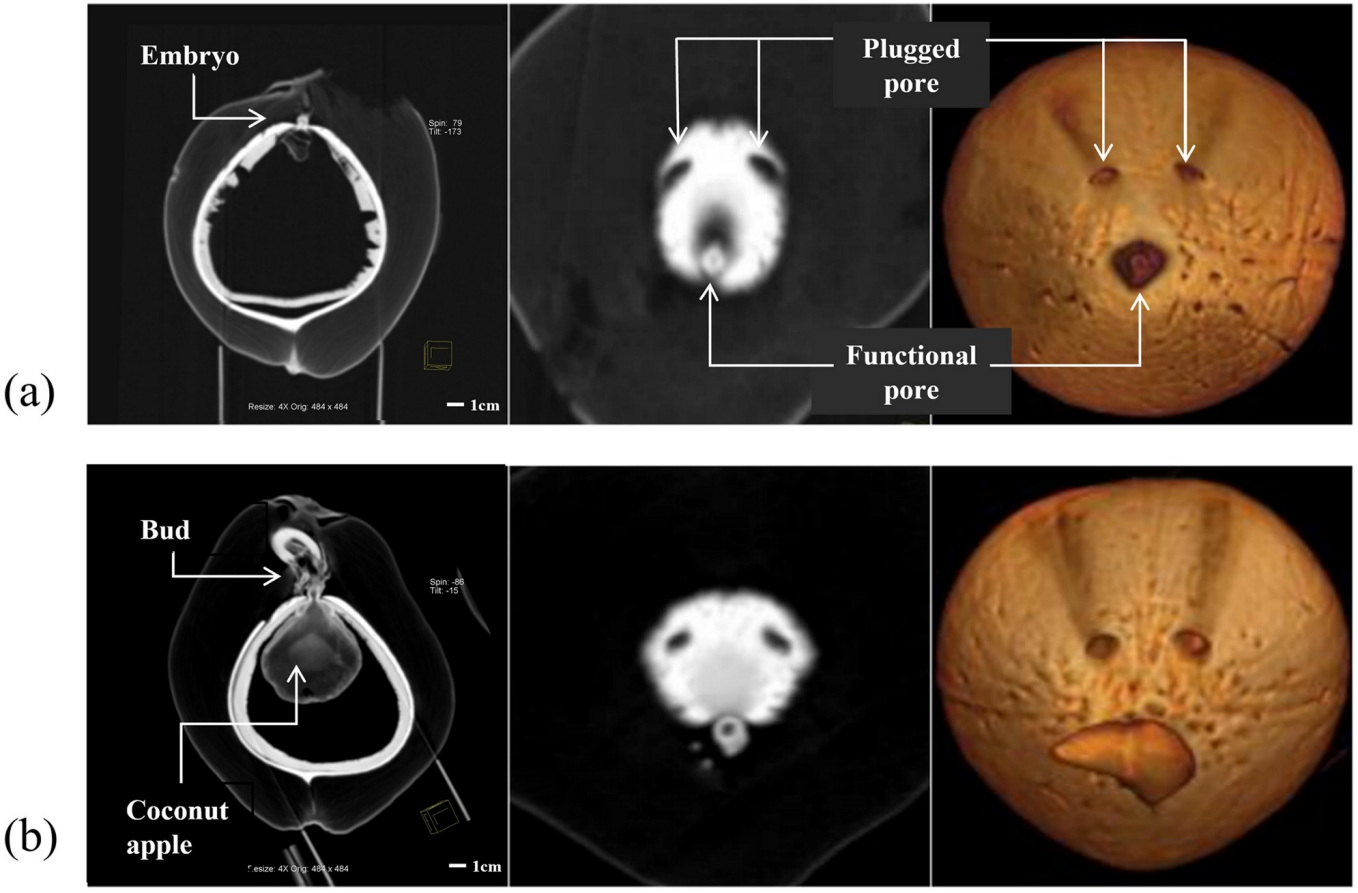
The structure of two coconuts (a, b). The multiplanar reformation on the left column show embryo, bud and coconut apples inside the coconut cavity. In the middle and right column, three pores on the surface of the endocarp and embryos sprouting from the largest pore.

In this study, the images were obtained by scanning 12 individual coconuts using CT for 3 months, once a week for the first two months and once every 2 weeks for the second month. MPR and VR were generated during the postprocessing. The MPR could clearly show the structures of the exocarp, mesocarp, endocarp, solid endosperm, liquid endosperm, bud, coconut apple, and roots on the two-dimensional cross-section plane (Fig 2). Different VR models were used to visualize the spatial relationship of the internal structures of the coconut. By assigning specific colors to the volumetric CT data of the exocarp and endocarp of coconut according to their CT values, we can create the VR models of the exocarp and endocarp respectively, and achieve simple visualization of the exocarp (i.e. the coconut appearance) and endocarp (Fig 3). Furthermore, by giving different colors and transparency to different structures’ voxel points, multiple internal structures can be displayed simultaneously, and the morphology and spatial relationship of the internal structures can be better observed (Fig 4). In addition, A combination of the VR and MPR may provide more information (Fig 5).

VR showed the internal structure of the coconut, such as the morphology of the coconut apple, endosperm, and bud. Particularly, we observed three pores in the endocarp, one of which was large and the other two pores were small, which looked similar to the shape of the eyes and mouth on a human face. It was observed that the embryos sprouted from the larger hole; however, no embryo or bud were observed in the other two pores. It was inferred that the largest pore on the endocarp surface served as the main channel for the uptake of nutrients by the growing sprouts of the coconut apple. On the first scan, CT images showed one coconut bud in the mesocarp and one bud visible in the outer pericarp. The germination rate of the coconuts in this sample was 50%, and 58.33% of the coconuts had coconut apples. The coconut morphology did not change with time. In coconuts without coconut apples, no germination was observed; only the internal structure of the coconut could be observed.

**Fig 6:**
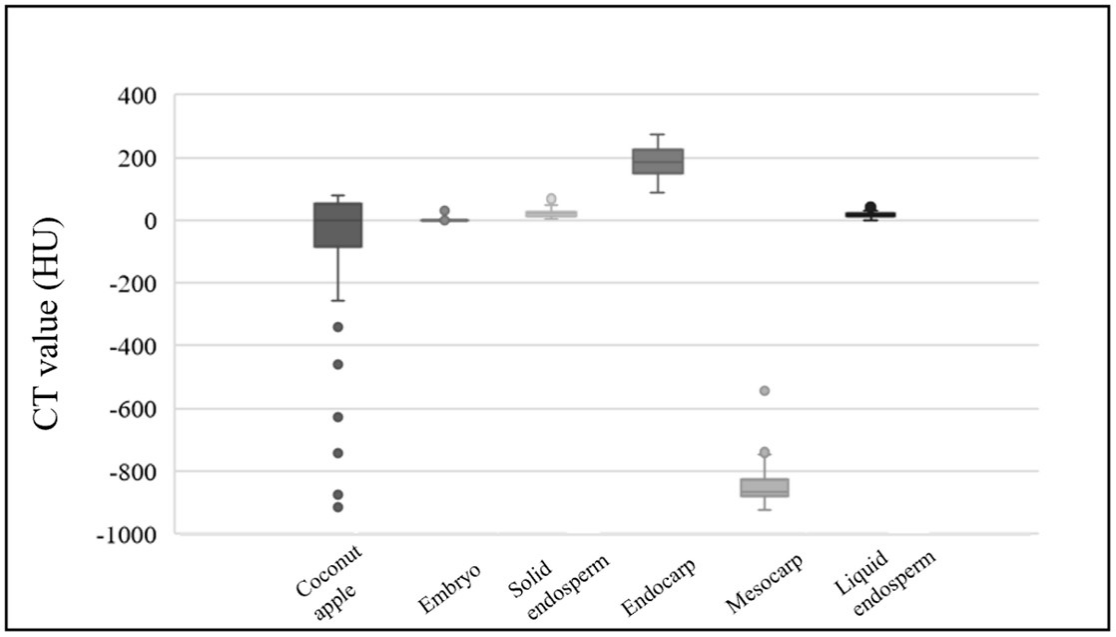
Computed tomography values for each component of coconuts.

**Fig 7:**
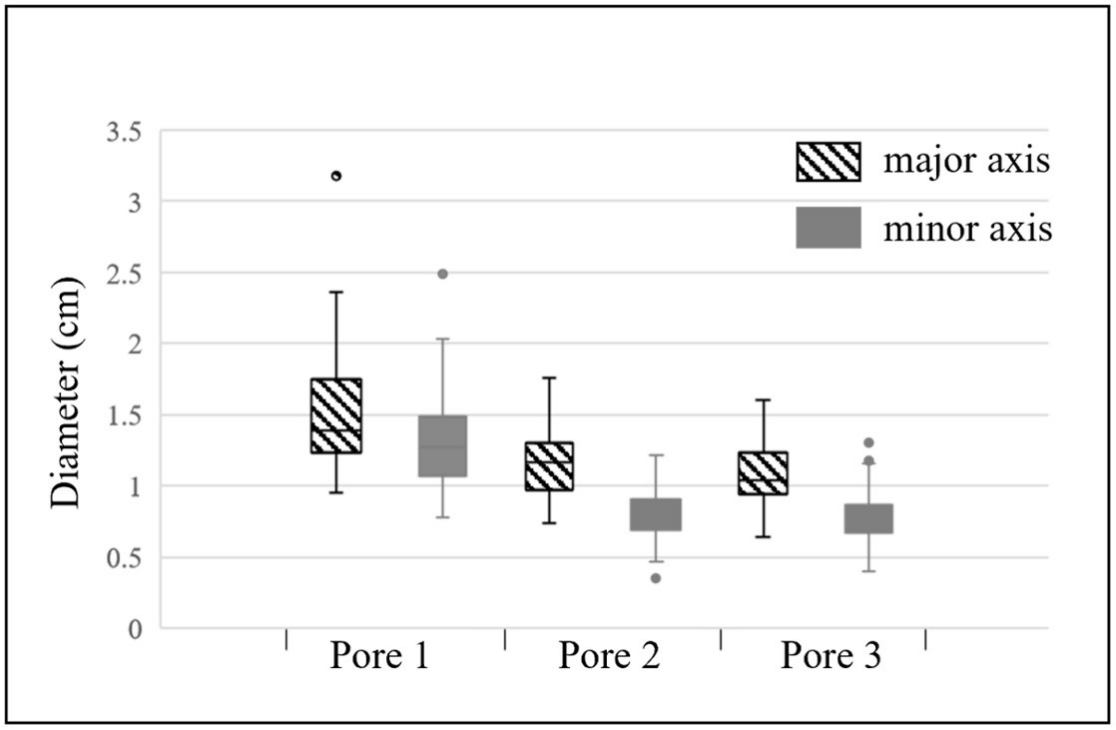
Measurements of the three pores on the coconut endocarp.

There was no significant change in CT value of each structure except bud and coconut apple (p > 0.05). The CT values of the 12 coconuts at four time points (with a 4-week interval) were collected and analyzed, and boxplots were drawn to understand their numerical distribution (Fig 6). The CT values of the coconut apple were sparsely distributed: −1000 to 100 HU; the CT values of the embryo ranged from 0 to 100 HU; the CT values of the solid endosperm ranged from −10 to 50 HU; the CT values of the liquid endosperm ranged from 0 to 60 HU; the CT values of the endocarp ranged from 130 to 300 HU; and the CT values of the mesocarp ranged from −1000 to −800 HU. In terms of morphological information, except for the weight of coconut and the volume of liquid endosperm, other parameters did not change significantly during the three months, including the size of coconut, the thickness of exocarp, mesocarp and endocarp, and the diameter of the three pores on the endocarp. Among them, an interesting pattern appeared in the diameter of the three poles of the endocarp: one pore was significantly larger than the other two pores, while the other two pores were almost the same size, and all three pores were oval in shape, as shown in Fig 7.

**Fig 8:**
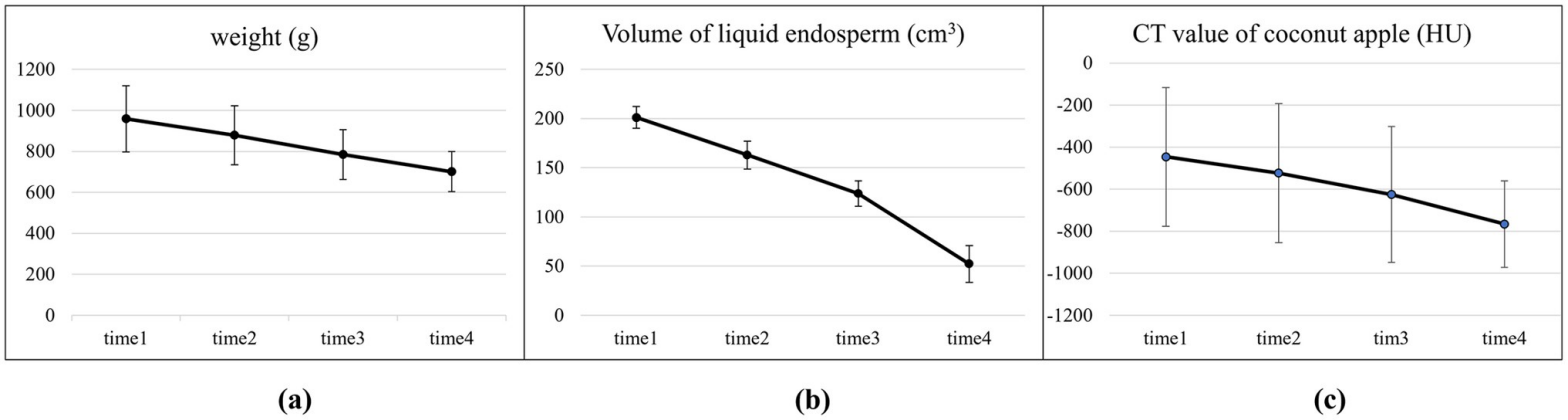
Coconuts changes observed over three months.

Among the parameters that changed significantly during the three months, a pairwise comparison was made between two adjacent time points, and the results showed that the weight and the liquid endosperm of the coconut had significant differences between all time points, while the CT value of the coconut apple only had significant differences between time 2 and time 3. The mean and standard deviation of the above three parameters at four time points (4 weeks interval) are shown in Fig 8. The Fig showed that coconut weight and the volume of liquid endosperm decreased significantly over three months, and the CT value of coconut apple has a slight decrease, with the change being more pronounced between time2 and time3 than other times.

**Fig 9:**
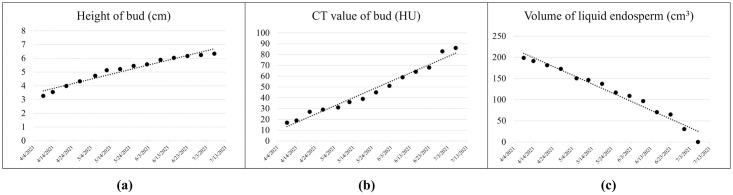
Measurements of a sprouted coconut observed over 3 months.

Due to the different growth stages of coconuts during the observation period, a coconut with a complete germination process was selected for analysis. A small bud was initially observed in this coconut. After 3 months, the liquid endosperm disappeared and the height of the bud increased from 3.26 cm to 6.34 cm. The CT value of the bud increased gradually, indicating that the bud was growing and its composition was changing. The relationship between measured results and time is shown in Fig 9. It can be observed that these changes show a linear trend.

**Fig 10:**
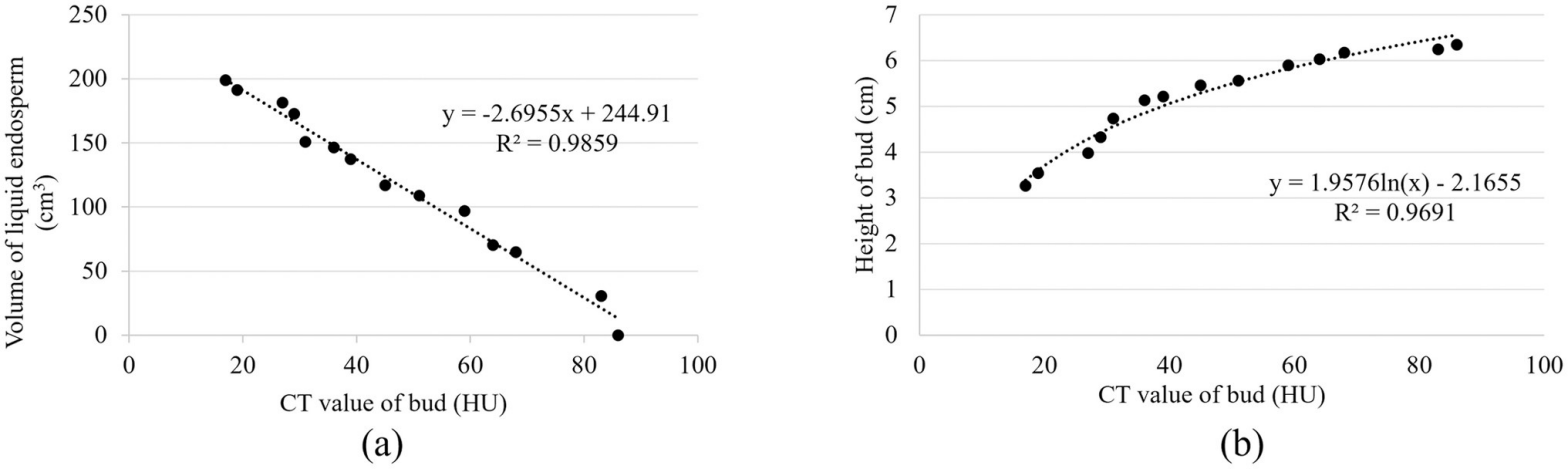
Regression analysis of the measurements of coconut.

Further regression analysis revealed a significant negative correlation between the CT value of the bud and the volume liquid endosperm, with the following regression equation: y = −2.6955x + 244.91; R^2^ = 0.9859 (Fig 10a). Moreover, a significant positive correlation was found between the height and CT value of the bud, using a nonlinear correlation to obtain the following regression equation: y = 1.9576ln(x) −2.1655; R^2^ = 0.9691 (Fig 10b).

## Discussion

In this study, CT was applied to non-destructively observe coconuts. The traditional gray-scale CT images were used to measure the CT value of each structure, and the VR models and MPR images generated from CT axial images were used to observe the morphological features of the internal structures of the coconut. The morphological and component changes in the exocarp, mesocarp, endocarp, solid endosperm, liquid endosperm, embryo, and bud were analyzed over three months. The VR models and MPR images can clearly and intuitively showed the spatial relationship between structures, such as the location of coconut apple, bud and endocarp, and the morphological changes of the embryo and bud during germination. We found that the endocarp surface has three pores with an appearance similar to that of the eyes and mouth of the human face, and the bud sprouted from the largest pore. In addition, we found that CT values of coconut apple and buds, liquid endosperm volume, and coconut weight changed significantly over 3 months.

In a previous study, CT images of plant roots in the soil can obtain the 3D visual information of root system, which can be used to perform genetic analysis of plant phenotypic correlation [21]. A study took X-rays of walnut shell structures to analyze structural changes [22], which are ideas that can provide reference for studying coconuts. Because the mesocarp of coconuts is thick and the endocarp is extremely hard, observing the growth of coconut bud is far less convenient than observing those of other plants; thus, medical CT may be a valuable auxiliary tool. Most studies on coconut structure were based on microscopic imaging technology, the mechanical performance of coconut endocarp was analyzed to improve the toughness of the composite materials [7]. Medical CT imaging, if combined with microscopic level information, can help design the structure of biomaterials.

In this experiment, we tracked the growth of coconuts for 3 months and recorded the germination process of one coconut. The results showed that we successfully tracked the germination process of a coconut, which means that the observation of coconuts using CT is feasible, and this technique is a potential tool for coconut research. The statistical results of this study showed that with the growth of the embryo, the CT value of the embryo increased, the volume of coconut water decreased, and the weight of coconut slightly decreased. The regression formula could be used to express the growth process, and the fitting accuracy was good, indicating that this technique can be applied to non-destructively observe the entire growth of coconuts and provides valuable information for coconut seed and fruit screening. Additionally, CT provides quantitative information on the structural components of coconuts at different growth stages, which can be associated with the physicochemical properties and nutritional value of coconuts [23–25].

The present study has several limitations. Only 12 coconuts were analyzed in this study, with a germination rate of 50%. Numerous samples are required to further explore. In addition, whether multiple CT scans on coconuts have a negative effect on the germination process remains unclear, and controlled trials are needed. In the future, the morphological and composition changes in coconuts over time can be further analyzed and examined. Additionally, the comparison of the structure and development of different varieties of coconuts needs further exploration.

## Conclusion

This article explored the feasibility of medical CT imaging technology for analyzing the structure of coconut and the changes during germination. Some interesting changes in the coconuts during the three months of the experiment were observed with the use of the visualization model and quantitative data obtained by CT technology.

## Supporting information

S1 Data (XLSX)
